# Serum Metabolomic Profiling of Patients with Lipedema

**DOI:** 10.3390/ijms242417437

**Published:** 2023-12-13

**Authors:** Sally Kempa, Christa Buechler, Bandik Föh, Oliver Felthaus, Lukas Prantl, Ulrich L. Günther, Martina Müller, Stefanie Derer-Petersen, Christian Sina, Franziska Schmelter, Hauke C. Tews

**Affiliations:** 1 Department of Plastic, Hand, and Reconstructive Surgery, University Hospital Regensburg, 93053 Regensburg, Germany; 2 Department of Internal Medicine I, Gastroenterology, Hepatology, Endocrinology, Rheumatology, and Infectious Diseases, University Hospital Regensburg, 93053 Regensburg, Germany; 3 Institute of Nutritional Medicine, University Medical Center Schleswig-Holstein, Campus Lübeck, 23538 Lübeck, Germany; 4 Department of Medicine I, University Medical Center Schleswig-Holstein, Campus Lübeck, 23538 Lübeck, Germany; 5 Institute of Chemistry and Metabolomics, University of Lübeck, 23562 Lübeck, Germany; 6 Fraunhofer Research Institution for Individualized and Cell-Based Medical Engineering (IMTE), 23562 Lübeck, Germany

**Keywords:** metabolomics, lipedema, ^1^H-nuclear magnetic resonance spectroscopy, ^1^H-NMR, adipose tissue

## Abstract

Lipedema is a chronic condition characterized by disproportionate and symmetrical enlargement of adipose tissue, predominantly affecting the lower limbs of women. This study investigated the use of metabolomics in lipedema research, with the objective of identifying complex metabolic disturbances and potential biomarkers for early detection, prognosis, and treatment strategies. The study group (n = 25) comprised women diagnosed with lipedema. The controls were 25 lean women and 25 obese females, both matched for age. In the patients with lipedema, there were notable changes in the metabolite parameters. Specifically, lower levels of histidine and phenylalanine were observed, whereas pyruvic acid was elevated compared with the weight controls. The receiver operating characteristic (ROC) curves for the diagnostic accuracy of histidine, phenylalanine, and pyruvic acid concentrations in distinguishing between patients with lipedema and those with obesity but without lipedema revealed good diagnostic ability for all parameters, with pyruvic acid being the most promising (area under the curve (AUC): 0.9992). Subgroup analysis within matched body mass index (BMI) ranges (30.0 to 39.9 kg/m^2^) further revealed that differences in pyruvic acid, phenylalanine, and histidine levels are likely linked to lipedema pathology rather than BMI variations. Changes in low-density lipoprotein (LDL)-6 TG levels and significant reductions in various LDL-2-carried lipids of patients with lipedema, compared with the lean controls, were observed. However, these lipids were similar between the lipedema patients and the obese controls, suggesting that these alterations are related to adiposity. Metabolomics is a valuable tool for investigating lipedema, offering a comprehensive view of metabolic changes and insights into lipedema’s underlying mechanisms.

## 1. Introduction

Lipedema is a chronic condition characterized by disproportionate and symmetrical enlargement of adipose tissue, predominantly affecting the lower limbs. It is often accompanied by pain, tenderness, and easy bruising, significantly impacting the quality of life of affected individuals [1]. Lipedema mainly affects women, typically starting during stages of hormonal change such as puberty, pregnancy, in vitro fertilization, and menopause [2].

The lipedema pathogenesis remains poorly understood, and its etiology and pathophysiological mechanisms are far from clarified [3,4,5,6]. Consequently, effective diagnostic and treatment strategies are limited. Usually, the condition is diagnosed clinically on the basis of history and physical examination results. Affected women are often first confronted with the diagnosis of obesity and advised on the usual weight-reducing measures. Because there is no specific test or biomarker for this disease, years often pass before lipedema is first diagnosed, leading to psychosocial distress, including depression, eating disorders, and social isolation [1,7].

In recent years, there has been growing interest in the application of metabolomics, a powerful and comprehensive analytical approach, to investigate the metabolic alterations associated with various diseases [8], including lipedema [2].

To investigate the altered metabolic pathways and to identify potential biomarkers that contribute to lipedema development and progression, we compared the metabolic profiles of female patients with lipedema with a lean as well as an obese control group. By identifying distinct metabolic profiles and subtypes within the lipedema population, the provided data may facilitate the development of targeted interventions and treatment strategies tailored to individual patients. This precision medicinal approach may enhance therapeutic outcomes and improve the overall management of patients with lipedema.

## 2. Results

### 2.1. Clinical Characteristics of the Study Cohort

A total of 75 women were enrolled in this study. The clinical and demographic features of the participants are summarized in Table 1. Among the 25 patients with lipedema, three presented with stage I, seventeen with stage II, and five with stage III lipedema. Most women with lipedema reported a family history of this condition (80%). The onset of lipedema most often occurred during puberty (48%).

### 2.2. Serum Metabolic and Lipidomic Profiles

**Figure 1:**
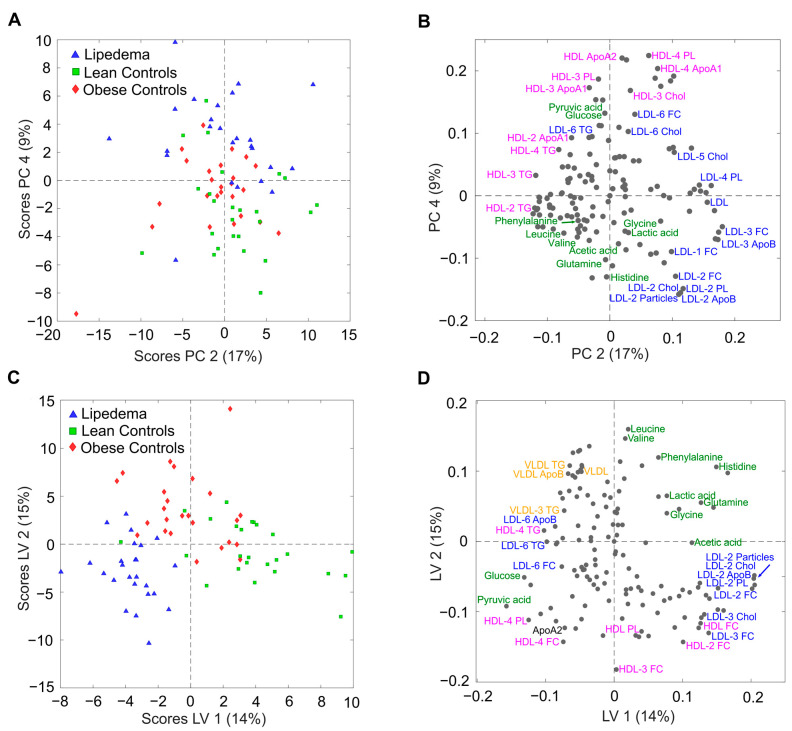
Multivariate statistical analysis of serum metabolic and lipidomic profiles between lipedema patients and nonlipedema controls. (**A**) Score plots of principal component analysis (PCA) and (**C**) partial least square discriminant analysis (PLS-DA), showing a distinct separation between lipedema samples (blue triangles), lean controls (green squares), and obese controls (red diamonds). The corresponding loading plots (**B**,**D**) display a selection of metabolites and lipoproteins contributing to this clustering. The parameters are color-coded according to their substance classes (metabolites: green, VLDL: orange, LDL: blue, HDL: pink, others: black). PC: principle component; LC: latent variable.

Unsupervised principal component analysis (PCA) (Figure 1A,B) of the quantified in vitro diagnostic research (IVDr) data was performed to visualize the entire dataset and identify the presence of outliers. Furthermore, a supervised partial least squares discriminant analysis (PLS-DA) model was constructed and revealed a notable distinction between the lipedema patients (red diamonds), lean controls (green squares), and obese controls (blue triangles) (Figure 1C,D).

The PCA score plot (Figure 1A) shows a weak separation between the three groups in principal component (PC) 2 vs. PC4, whereas the corresponding loading plot (Figure 1B) highlights the selection of relevant metabolites and lipoprotein parameters contributing to this clustering. Since PCA is an unsupervised method that was used to analyze the variance in the entire dataset, we performed further supervised PLS-DA to focus more on the patient groups of interest and highlight the specific differences between them. The score plot of the PLS-DA model (Figure 1C) shows a stronger separation between the lipedema group and the two weight control groups. Clustering was internally validated through reliable cross-validation (CV) (96% for lipedema, 85% for lean controls, and 74% for obese controls) from the areas under the receiver operating characteristic (AUROC) curves. The loading plot indicates that alterations in the contents of several amino acids, glucose, carboxylic acids, and low-density lipoprotein (LDL) particles contributed to the good separation of the three cohorts (Figure 1D).

**Figure 2:**
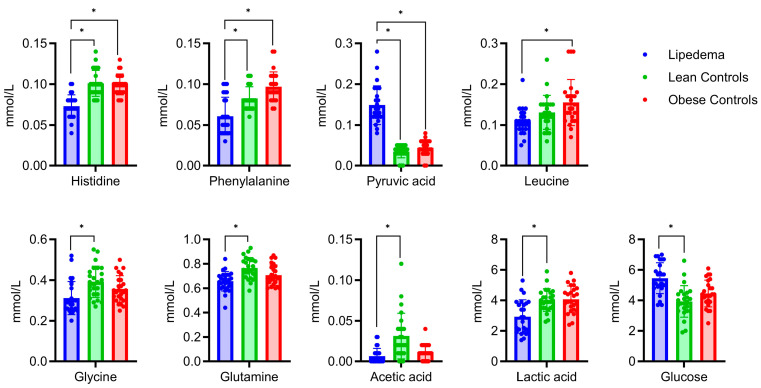
Specific metabolites of lipedema patients compared with weight controls. Significant differences between the groups (lipedema (blue, left), lean controls (green, middle), and obese controls (red, right)) were identified with the Mann–Whitney test (*p* < 0.05; false discovery rate (FDR): 1%) and labeled with a star (*).

Multiple Mann–Whitney tests identified metabolic differences between the patients with lipedema, those with normal weight, and the obese controls (*p* < 0.05; false discovery rate (FDR): 1%) (Figure 2). First, the differential metabolites included histidine and phenylalanine, which were lower in the lipedema group, and pyruvic acid, which was higher in the lipedema group than in both control groups. Second, the concentration of leucine was lower in the lipedema group than in the obese controls. Third, we observed that the acetic acid, glutamine, glycine, and lactic acid levels were lower and the glucose levels were higher in the lipedema group than in the lean controls. The full list of annotated metabolites is presented in Supplementary Table S1.

**Figure 3:**
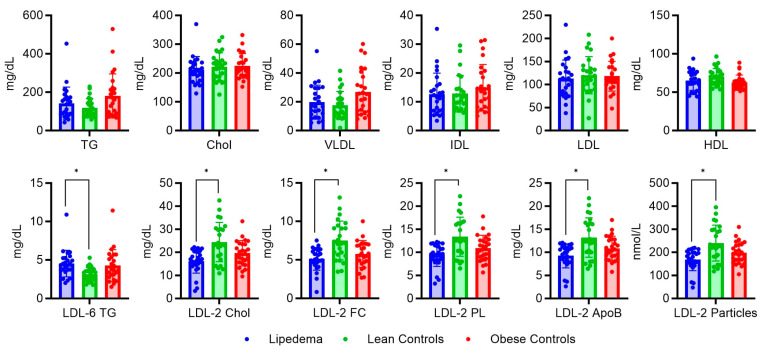
Standard lipid parameters and significantly altered low-density lipoprotein (LDL) subfractions of patients with lipedema compared with lean and obese controls. For classical lipid parameters, no significant differences between lipedema patients (blue, left), lean controls (green, middle), and obese controls (red, right) were observed. For specific subfractions, the Mann–Whitney test (*p* < 0.05; FDR: 1%) was used to identify differences between the lipedema patients and the lean controls. Significant differences are labeled with a star (*). TG: triglyceride; Chol: Cholesterol; VLDL: very low-density lipoprotein, IDL: intermediate-density lipoprotein; HDL: high-density lipoprotein; Chol: cholesterol; FC: free cholesterol; PL: phospholipid; ApoB: apolipoprotein B.

Regarding the lipid profiles, we did not observe differences in the standard parameters such as LDL or the total serum cholesterol levels of the three groups (*p* < 0.05; FDR: 1%) (Figure 3). In-depth analysis focused on examining alterations in specific lipid subfractions. Among the findings, it was observed that LDL-6 triglyceride (TG) levels were elevated in individuals with lipedema. Additionally, lipedema patients exhibited significant reductions in several LDL-2 subfractions, including LDL-2 cholesterol (Chol), LDL-2 phospholipids (PL), LDL-2 TG, and LDL-2 free cholesterol (FC), when compared with individuals of normal weight.

### 2.3. Analysis of Metabolite Variations in Relation to Body Mass Index: Subgroup Comparison and Correlation Analysis

**Figure 4:**
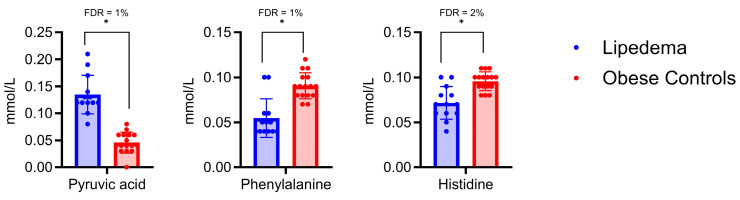
Matched subgroup analysis of metabolite levels in lipedema patients and obese controls within a BMI range of 30.0 to 39.9 kg/m^2^ (obesity classes I and II). Significant differences between the groups (lipedema (blue, left) and obese controls (red, right)) were identified with the Mann–Whitney test (*p* < 0.05; FDR: see legend) and labeled with a star (*). Pyruvic acid, phenylalanine, and histidine levels remained significantly different between the lipedema and obese control groups.

To better isolate the effects of lipedema from those of the body mass index (BMI), we created matched subgroups within our study population to compare the patients with lipedema (n = 13) with the obese controls (n = 16) within a BMI range of 30.0 to 39.9 kg/m^2^ (obesity class I and class II). This strategy allowed for a more direct comparison by minimizing the confounding effect of the BMI. Upon reanalysis, the pyruvic acid, phenylalanine, and histidine levels remained significantly different between the lipedema and obese control groups, suggesting that these differences are likely related to the disease pathology rather than BMI variations (Figure 4). Leucine, however, did not show significant differences.

**Figure 5:**
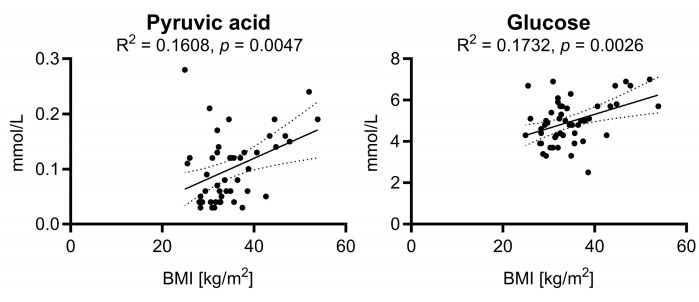
Analysis of correlation between BMI and the relevant metabolites in all overweight subjects. Of the significant parameters (see Figure 2) only pyruvic acid and glucose showed weak correlations with the BMI.

Furthermore, we performed a correlation analysis between the BMI and the relevant metabolites in all subjects (Supplementary Table S2). That analysis specifically focused on how the BMI correlates with various parameters that were identified as significant in this study. Of the parameters analyzed, only pyruvic acid and glucose showed weak correlations with the BMI (Figure 5).

### 2.4. Diagnostic Accuracy of Pyruvic Acid, Histidine, and Phenylalanine in Predicting Lipedema

**Figure 6:**
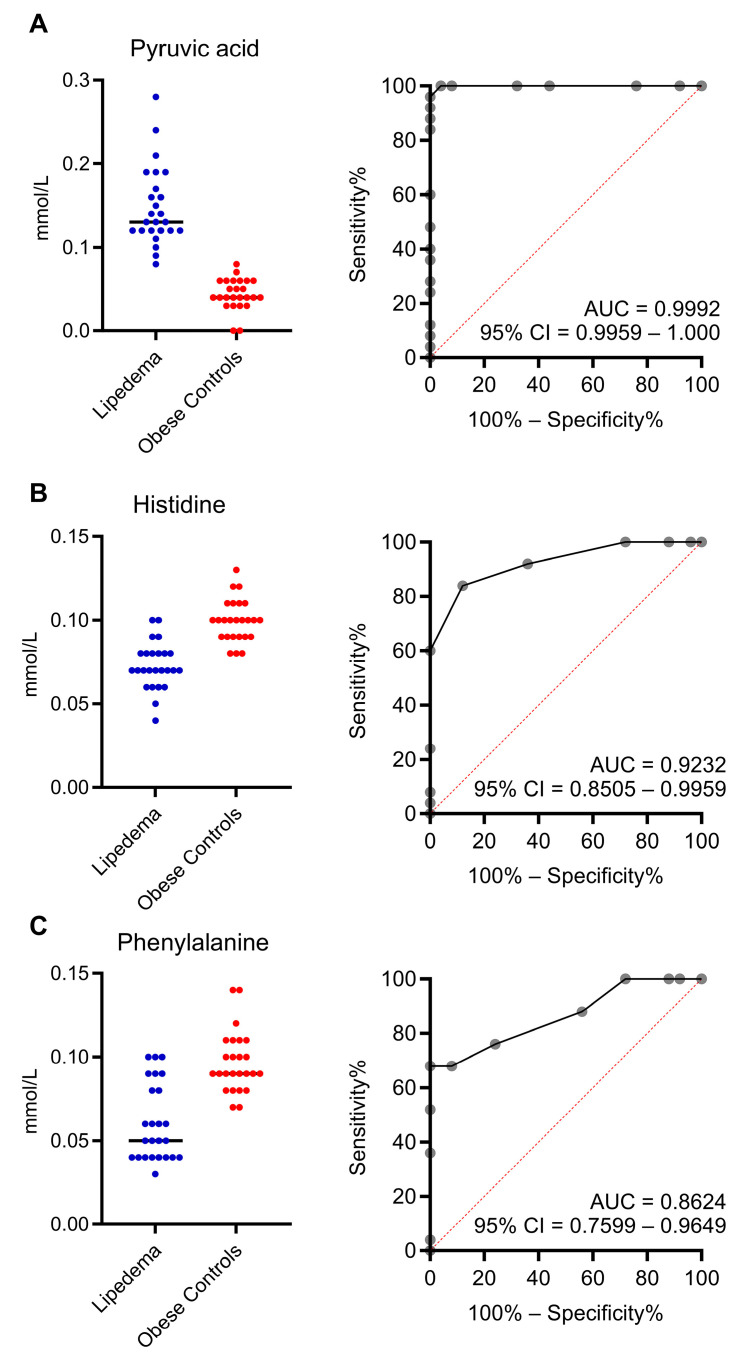
Receiver operating characteristic (ROC) curves used to test the diagnostic performances of the selected metabolites. Differences in the serum levels of pyruvic acid (**A**), histidine (**B**), and phenylalanine (**C**) are shown between lipedema patients (blue, left) and obese controls (red, right). The ROC curves, corresponding areas under the curves (AUCs), and 95% confidence intervals (CIs) indicate diagnostic potential.

The receiver operating characteristic (ROC) curve for the diagnostic accuracies of pyruvic acid, histidine, and phenylalanine for lipedema is presented in Figure 6. All parameters had good diagnostic ability, with pyruvic acid being the most promising. The area under the curve (AUC) of pyruvic acid was 0.9992, with a 95% confidence interval (CI) between 0.9959 and 1.000. The AUC of histidine was 0.9232 (95% CI = 0.8505–0.9959), and that of phenylalanine was 0.8624 (95% CI = 0.7599–0.9649). For pyruvate levels with an optimal cutoff value of >0.075 mmol/L, the sensitivity and specificity of the diagnosis of lipedema were 1.00 and 0.98, respectively.

## 3. Discussion

The main findings from this study show that the metabolic and lipidomic profiles of patients with lipedema differ significantly from those of sex- and age-matched lean and obese controls without lipedema. Of the 39 metabolites, nine were significantly altered in lipedema. To the best of our knowledge, this study is the first to address the potential use of ^1^H-nuclear magnetic resonance (^1^H-NMR) spectroscopy in the analysis of serum from patients with lipedema, unlike others that have analyzed serum metabolites with liquid chromatography-mass spectrometry (LC-MS). Our results are consistent with those of some but not all previous studies [2,9,10]. Felmerer et al. [9] demonstrated slight differences in the systemic lipid metabolism in lipedema, with systemic lipid values ranging between the upper physiological range and the slight pathological range (increased levels of total cholesterol: 1.31-fold; LDL: 1.46-fold; and TGs: 1.49-fold, with a 1.37-fold increase in apolipoprotein B compared with age- and sex-specific standardized physiological ranges). HDL and apolipoprotein A were found to be comparable between the two groups. Unlike in our approach, Felmerer et al. analyzed the fasting serum samples and the oily phases of lipoaspirates obtained from 10 patients with lipedema (one at stage I, two at stage II, and seven at stage III) and 11 gender- and BMI-matched control patients. Wolf et al. [11] conducted a lipidomic analysis comparing adipose tissue and serum between individuals with lipedema (n = 20) and anatomically and BMI-matched control participants (n = 10). Those findings revealed that there were no notable alterations in lipid composition in either of the tissue groups. This is in accordance with our observation that altered lipoprotein levels and LDL composition are related to adiposity rather than lipedema. In contrast, Nankam et al. [10] found higher concentrations of total cholesterol and LDL-C in patients with lipedema than in BMI-matched controls. Ishaq et al. [2] compared the metabolic and lipid profiles of adipocytes from BMI-matched patients with lipedema (n = 4) and unaffected volunteers (n = 4). The leading significantly increased lipids in lipedema included glycerophospholipids (466 molecules). Metabolic pathway analysis has suggested that perturbations in lipedema include pathways linked to amino acid metabolism (lysine biosynthesis and glutamate metabolism), fatty acyls, and glycerophospholipids.

From a metabolic perspective, the differential metabolites identified in our study can provide valuable insights into the metabolic alterations associated with lipedema. Histidine and phenylalanine are amino acids that are crucial in various metabolic pathways. Their lower levels in the lipedema group may indicate alterations in protein metabolism, amino acid utilization, and related pathways. These amino acids are the precursors to various essential molecules, and their perturbations could influence processes such as protein synthesis, energy production, and neurotransmitter regulation. These changes suggest potential disruptions in amino acid metabolism in patients with lipedema. Pyruvate is a central molecule in adipocyte metabolism [12], contributing to energy production, triglyceride synthesis, fatty acid production, and glucose regulation [13,14]. While in our study, pyruvate levels weakly correlated with the BMI, lipedema might exacerbate this increase independently of the BMI. Both high BMIs and lipedema might independently contribute to increased pyruvate levels, with lipedema having an additive or synergistic effect. Its metabolism is tightly regulated to meet the energy and storage demands of adipocytes in response to nutritional and physiological cues. In adipocytes, disrupted pyruvate metabolism results in the accumulation of triglycerides [15]. Although pyruvate is not a direct precursor to triglyceride synthesis, the availability of acetyl-CoA (derived from pyruvate) is essential for de novo fatty acid synthesis [16]. This could be linked to the observed metabolic changes in lipedema. Elevated pyruvate levels in lipedema may indicate a disturbance in the citric acid cycle. While pyruvate is a classic glycolytic metabolite, its accumulation may suggest a disruption of downstream metabolic pathways. If pyruvate does not efficiently enter the Krebs cycle, it may accumulate, leading to high levels. The decreased levels of acetic acid, glycine, glutamine, and lactic acid in the lipedema group suggest perturbations in multiple metabolic pathways. Acetic acid reduction could be indicative of altered lipid metabolism, particularly fatty acid oxidation or synthesis [17]. There is evidence that acetic acid has a role in body weight control and adipose tissue function [18], but the long-term effects of acetate in humans have not been investigated, to the best of our knowledge. Serum glycine levels have been found to be positively correlated with subcutaneous fat mass and negatively correlated with abdominal adiposity [19]. This suggests a role of glycine in the distribution of adipose tissue. Studies in animal models of obesity and type 2 diabetes have shown that glycine supplementation can reduce white fat and an improve insulin sensitivity [20]. These findings prompt the question of whether glycine supplementation could serve as a therapeutic approach for patients with lipedema. Understanding the connections between glycine, one-carbon metabolism, glycolysis, and histidine metabolism can offer a deeper insight into the metabolic intricacies of lipedema. A hyperglycolytic phenotype, for instance, might enhance the production of serine and glycine. Glutamine is involved in nitrogen metabolism [21], energy production [22], and neurotransmitter synthesis [23]. The decrease thereof may reflect changes in these processes. Glutamine may lower adipose tissue mass and the inflammation of fat tissues [24]. Lower lactic acid levels may indicate altered glycolytic or oxidative metabolism. Lactic acid has multiple functions and was found to induce the beiging of adipocytes [25]. Beige fat cells express high levels of uncoupling protein 1 and produce heat by burning off fat [26]. Initially, the leucine levels were lower in the lipedema group than in the obese controls, hinting at a metabolic difference related to lipedema. However, further examination of BMI-matched groups showed no significant differences in the leucine levels, suggesting that other factors, possibly individual metabolic variations, might explain the initial observation. This highlights the complexity of metabolic interactions in obesity and lipedema and calls for further research to understand leucine metabolism in these conditions, considering factors beyond BMI.

The observed metabolic alterations may reflect a combination of factors, including disrupted amino acid metabolism, altered glycolysis, and the citric acid cycle. Lipedema is characterized by abnormal fat accumulation, and metabolic changes could be related to adipose tissue dysfunction, inflammation, and altered energy homeostasis. These changes could contribute to the pathogenesis of lipedema by influencing fat storage in adipose tissues and peripheral organs, tissue inflammation, and overall metabolic regulation. Our focus on metabolomics complements recent research efforts that have investigated diagnostic and biomarker tools on the cellular and genetic levels [2,27], emphasizing the multifaceted nature of lipedema.

This study identified differences in circulating markers between lipedema patients, lean controls, and obese controls but faced limitations due to the heterogeneity in metabolic health within our lipedema subgroup. Addressing this heterogeneity and ensuring a more uniform metabolic health status across subgroups will be crucial for future research. We believe that these findings represent a valuable addition to the current state of knowledge and could guide future studies to elucidate the pathophysiology of lipedema.

NMR spectroscopy is a nondestructive technique that requires minimal sample preparation, offers insights into key metabolic pathways, and sets the stage for exploring the impact of dietary modifications [28,29,30] and the introduction of nutritional supplements [31,32] through longitudinal metabolomic approaches. Some critical gaps and challenges still need to be addressed to fully leverage its potential, including the need for standardized protocols for sample collection. Age and BMI differences are vital factors in metabolomic studies [33], with specific metabolites closely linked to the BMI, such as branched-chain amino acids [34]. Furthermore, it is essential to integrate metabolomic data with other -omic data, such as those of genomics, transcriptomics, and proteomics, to comprehensively understand biological processes and disease mechanisms.

## 4. Materials and Methods

### 4.1. Study Design and Ethical Approval

This case–control study was approved by the local Ethics Committee, and all participants provided informed written consent. Formal and documented ethical approval was obtained (reference number 22-3163-101, University of Regensburg, and reference number Nr. 22-104, University of Lübeck). A total of 75 subjects were enrolled in this study.

### 4.2. Selection of Lipedema Patients

Twenty-five patients with lipedema were included, following the German S1 guidelines [35]. The characteristics of the patients with lipedema are listed in Table 2. The participants in this group were carefully selected on the basis of clinical evaluations and included individuals from all stages of lipedema. Clinical examination and conventional color Doppler flow imaging (CDFI) excluded other common causes of leg swelling, such as chronic venous insufficiency and lymphedema. The clinical information of the patients with lipedema was collected, including age, BMI, lipedema stage, and clinical symptoms. Exclusion criteria included asymmetry of legs, a waist–hip ratio of >0.85, previous liposuctions, diseases that could affect examination quality (e.g., active infectious diseases), primary obesity lacking disproportion, and evidence of lipedema. In addition, patients with fat distribution disorders from different causes (e.g., painless lipohypertrophy, benign symmetrical lipomatosis, or lipomatosis dolorosa), participants with edema-causing conditions (e.g., lymphedema, phlebedema, or myxedema), and pregnant and lactating women were also ineligible for this study.

### 4.3. Selection of the Control Groups

Two control groups were created by matching healthy volunteers without lipedema by age and sex. For the control groups, we used the Luebeck Metabolomics Reference (LuMeR) cohort, which was initially developed for the Luebeck Longitudinal Investigation of SARS-CoV-2 Infection (ELISA) framework [36], recruited from the general healthy population. Using the World Health Organization (WHO) body mass index classification [37], individuals were classified as normal weight (BMI: 18.5–24.9 kg/m^2^) and overweight or obese (BMI > 24.9 kg/m^2^). The first control group (n = 25) consisted of volunteers with BMIs over 24.9 kg/m^2^ and who were matched for age and sex but did not have lipedema, chronic diseases, or pregnancy. We defined chronic diseases as conditions with a duration of at least one year that require ongoing medical attention, limit the activities of daily living, or both, including cancer; diseases of the nervous system (multiple sclerosis, Parkinson’s disease, stroke); diseases of the respiratory system (asthma, chronic obstructive pulmonary disease); diseases of the cardiovascular system (coronary heart disease, cardiac insufficiency, high blood pressure, condition following a heart attack); diseases of the digestive system (irritable bowel syndrome, Crohn’s disease, ulcerative colitis); diseases of the liver (liver cirrhosis, elevated blood lipid levels); diseases of the urogenital system (chronic kidney failure, chronic cystitis); diseases of metabolism (diabetes, hypothyroidism); diseases of the skin (psoriasis); diseases of the skeletal system, muscles, and connective tissue (chronic polyarthritis, ankylosing spondylitis, rheumatic muscle inflammation, osteoporosis); and mental health conditions (depression). The second control group (n = 25) comprised volunteers with normal weights (BMI between 18.5 and 24.9 kg/m^2^) and no lipedema diagnosis, matched for age and sex. Similarly, to the obese control group, the participants in this group had no chronic diseases or pregnancies. 

### 4.4. Sample Collection, Preparation, and ^1^H NMR Spectroscopy

Nonfasting serum samples were collected, centrifuged 30 min after collection, and stored at −80 °C until ^1^H-NMR spectroscopy analysis. The samples were analyzed using Bruker’s standardized IVDr procedure (Bruker BioSpin, Ettlingen, Germany). The strict standard operating procedure has been described previously [38,39]. In brief, frozen aliquots were thawed at room temperature for several minutes. Serum and a phosphate buffer (75 mM; pH: 7.4; 20% deuterium oxide; 4.8 mM 3-trimethyl-silyl-[2,2,3,3-^2^H_4_]propionic acid (TSP-d4)) were homogenized, and 600 μL of the well-mixed sample was transferred to a 5 mm NMR tube. The tubes were cooled at 279 K in an automated SampleJet^TM^ (Bruker, Ettlingen, Germany) until measurement. The samples were analyzed using a 600 MHz Avance III HD NMR spectrometer with a TXI probe at 310 K. A standard operating procedure (SOP) was performed prior to analysis, including checking of temperature calibration, quantification, and water suppression performance. A one-dimensional (1D) NOESY experiment (pulse program: noesygppr1d) and a 1D Carr–Purcell–Meiboom–Gill spin-echo experiment (CPMG) (pulse program: cpmgpr1d) for the suppression of proteins and other macromolecular signals were recorded per sample. Furthermore, 2D J-resolved and diffusion-edited experiments were performed. Bruker Quantification in Plasma/Serum (B.I.Quant-PS 2.0.0) and Bruker IVDr Lipoprotein Subclass Analysis (B.I.-LISA) were used to automatically quantify 39 metabolites (+2 technical additives) and 112 lipoprotein parameters (Bruker BioSpin), including very-low-density lipoprotein (VLDL), intermediate-density lipoprotein (IDL), low-density lipoprotein (LDL), and high-density lipoprotein (HDL). In addition, several subfractions of triglycerides (TG), cholesterol (Chol), free cholesterol (FC), phospholipids (PL), and apolipoproteins (Apo) were calculated. The LDL was categorized into six subclasses based on specific density ranges: LDL-1 (1.019–1.031), LDL-2 (1.031–1.034), LDL-3 (1.034–1.037), LDL-4 (1.037–1.040), LDL-5 (1.040–1.044), and LDL-6 (1.044–1.063).

### 4.5. Statistical Analysis

For unsupervised PCA and supervised PLS-DA, the PLS toolbox (Eigenvector Research, Inc., Manson, WA, USA) was employed. For the PCA, quantified IVDr data were variance-scaled and mean-centered. In addition, for the PLS-DA, orthogonal signal correction (OSC) was performed. The PLS-DA was calculated, followed by cross-validation using Venetian blinds, and the AUROC curve was computed. Univariate statistical analyses and simple linear regression analyses were performed using GraphPad Prism, version 10.0.2 (GraphPad Software, Boston, MA, USA). Univariate analysis was performed by applying an unpaired, nonparametric Mann–Whitney test followed by correction for multiple comparisons using the method of Benjamini, Krieger, and Yekutieli to control the false discovery rate (FDR). Furthermore, an analysis employing receiver operating characteristic (ROC) curves was conducted to evaluate the diagnostic potential in distinguishing between patients with and without lipedema. The optimal cutoff value for pyruvate was defined by the points representing the highest concomitant sensitivity and specificity provided by GraphPad Prism.

## 5. Conclusions

This study highlights the significant roles of metabolomics and lipidomics in advancing our understanding of lipedema. By identifying distinct metabolic changes in serum samples, including variations in histidine, phenylalanine, and pyruvic acid levels, it has shown their relevance in differentiating lipedema- from obesity-related metabolic changes. Further research with expanded cohorts and uniform protocols is essential to validate these findings and understand them in a broader metabolic context.

## Figures and Tables

**Table 1:** Patient and control characteristics.

Clinical Characteristics	Lipedema (Lip.) n = 25	Lean Controls (Lean) n = 25	Obese Controls (Obese) n = 25	*p*-Value
Male	0	0	0	-
Female	25	25	25	-
Age, Years	48.4 (11.1)	48.4 (11.0)	45.0 (12.1)	lip. vs. lean > 0.9999 lip vs. obese 0.3121
Body Mass Index (BMI), kg/m^2^	37.0 (8.0)	22.7 (1.4)	32.4 (3.7)	lip vs. lean < 0.0001 lip vs. obese 0.0116
Normal Weight: BMI of 18.5 to 24.9 kg/m^2^	1	25	0	-
Overweight: BMI of 25 to 29.9 kg/m^2^	3	0	8	0.4606
Moderate Obesity (Class I): BMI of 30 to 34.9 kg/m^2^	8	0	12	>0.9999
Severe Obesity (Class II): BMI of 35 to 39.9 kg/m^2^	5	0	4	0.9048
Very Severe Obesity (Class III): BMI of 40 kg/m^2^ or Greater	8	0	1	-
Lipedema Stage (St):				
St. 1	3	N.A.	N.A.	-
St. 2	17	N.A.	N.A.	
St. 3	5	N.A.	N.A.	
Onset of Lipedema:				
Puberty	12	N.A.	N.A.	-
Pregnancy	3	N.A.	N.A.	
Menopause	2	N.A.	N.A.	
Other	8	N.A.	N.A.	
Family History of Lipedema	20	N.A.	N.A.	-
Concomitant Disease:				
Arterial Hypertension	6	0	0	-
Diabetes, Type 2	1	0	0	
Hypothyroidism	7	0	0	

Values are mean (±standard deviation) or absolute. N.A.: not applicable.

**Table 2:** Inclusion criteria for patients with lipedema (German S1 guidelines).

Female
Bilateral and symmetrical enlargement of the limbs
Minimal pitting edema
Pain, tenderness on palpation
Mild bruising
Persistent enlargement after elevation of the extremities or weight loss
Negative Kaposi–Stemmer sign

## Data Availability

The derived data supporting the findings of this study are available from the corresponding authors upon reasonable request.

## Supplementary Materials

The following supporting information can be downloaded at: https://www.mdpi.com/article/10.3390/ijms242417437/s1.
