# Supplementary material for: Serum Metabolomic Profiling of Patients with Lipedema
Source: Int J Mol Sci. 2023 Dec 13;24(24):17437. doi: 10.3390/ijms242417437 (PMC10743543; doi:10.3390/ijms242417437)
Supplement: Supplementary file 1 [file ijms-24-17437-s001.zip › ijms-2748638-supplementary.pdf]

Supplementary Material

1 Supplementary Tables

Table S1: Results of the unpaired, nonparametric Mann-Whitney test with correction for multiple comparisons using the method of Benjamini, Krieger, and Yekutieli to control the false discovery rate (FDR). *P*- and *q*-values (adjusted *p*-values) for comparisons between lipedema patients and controls. Furthermore, a subgroup analysis of patients with lipedema (n = 13) and obese controls (n = 16) within a body mass index (BMI) range of 30.0–39.9 kg/m² was performed to minimize the confounding effect of BMI. Parameters without significant changes across all comparisons are indicated in italics. In the manuscript, parameters are specified in greater detail, which are also considered sufficient in terms of their quantification criteria within the in vitro diagnostic (IVDr) procedure.

	Lipedema vs. Lean Controls		Lipedema vs. Obese Controls		Lean Controls vs. Obese Controls		Subgroup BMI 30.0-39.9 kg/m² Lipedema vs. Obese Controls	
	p value	*q* value	*p* value	*q* value	*p* value	*q* value	*p* value	*q* value
Ethanol	0.489796	0.625113	0.489796	0.754791	>0.999999	>0.999999	0.448276	0.873941
Trimethylamine -N-oxide	>0.999999	0.884575	>0.999999	0.976993	>0.999999	>0.999999	>0.999999	0.990196
2-Aminobutyric acid	0.79894	0.825409	0.265696	0.555661	0.188561	0.359732	0.056596	0.411146
Alanine	0.454757	0.612026	0.04423	0.268972	0.351878	0.503478	0.062418	0.411146
**Asparagine**	<0.000001	<0.000001	0.000545	0.01163	0.004056	0.093665	0.027829	0.374260
Creatine	0.442951	0.605545	0.671572	0.888377	0.763566	0.921827	0.767850	0.990196
Creatinine	0.668356	0.762308	0.265183	0.555661	0.30278	0.472793	0.418268	0.866223
**Glutamic acid**	0.000271	0.003189	0.008719	0.100255	0.242506	0.425846	0.366920	0.834046
**Glutamine**	0.000048	0.000932	0.075879	0.307416	0.023024	0.111183	0.820100	0.990196

**Glycine**	0.000274	0.003189	0.009886	0.105559	0.09993	0.245115	0.056530	0.411146
**Histidine**	<0.000001	<0.000001	<0.000001	<0.000001	0.951582	>0.999999	0.000360	0.013641
Isoleucine	0.188563	0.359438	0.119954	0.388976	0.91106	>0.999999	0.450335	0.873941
**Leucine**	0.016596	0.072457	0.000391	0.009752	0.130443	0.283852	0.009212	0.232602
**Lysine**	0.000714	0.00537	0.003424	0.051179	0.579114	0.73959	0.002728	0.082654
**Methionine**	<0.000001	<0.000001	<0.000001	<0.000001	0.022747	0.111183	0.000021	0.001555
N,N-Dimethylglycine	>0.999999	0.884575	>0.999999	0.976993	>0.999999	>0.999999	>0.999999	0.990196
**Ornithine**	0.352161	0.539579	0.000082	0.002454	0.107598	0.258768	0.032637	0.374260
**Phenylalanine**	0.000871	0.006206	0.000002	0.000083	0.001877	0.057997	0.000158	0.008003
Proline	0.079338	0.223699	0.020538	0.163657	0.28851	0.472793	0.041996	0.374260
Sarcosine	0.667101	0.762308	0.744519	0.918516	0.412361	0.554105	>0.999999	0.990196
Threonine	0.145805	0.313227	0.236177	0.528528	0.019891	0.106586	0.888582	0.990196
Tyrosine	0.933615	0.884575	0.176976	0.490009	0.218764	0.402448	0.227774	0.780358
Valine	0.149647	0.314023	0.003979	0.054066	0.080965	0.208525	0.019858	0.334274
2-Hydroxybutyric acid	>0.999999	0.884575	>0.999999	0.976993	>0.999999	>0.999999	>0.999999	0.990196
**Acetic acid**	0.000012	0.000268	0.020802	0.163657	0.001385	0.053504	0.169101	0.750350

Citric acid	0.06879	0.206889	0.039644	0.25765	0.557275	0.723662	0.363821	0.834046
Formic acid	0.740345	0.792575	0.050308	0.27852	0.015389	0.106586	0.166559	0.750350
**Lactic acid**	0.000297	0.003189	0.000626	0.011696	0.873787	>0.999999	0.040936	0.374260
Succinic acid	0.109857	0.280528	0.229266	0.528528	>0.999999	>0.999999	0.203065	0.750350
Choline	>0.999999	0.884575	>0.999999	0.976993	>0.999999	>0.999999	>0.999999	0.990196
2-Oxoglutaric acid	0.489796	0.625113	0.489796	0.754791	>0.999999	>0.999999	0.192118	0.750350
3-Hydroxybutyric acid	0.457371	0.612026	0.479432	0.754791	0.066096	0.182389	0.951438	0.990196
Acetoacetic acid	0.063116	0.194139	0.076339	0.307416	0.983355	>0.999999	0.345004	0.834046
**Acetone**	0.000156	0.002637	0.01279	0.119492	0.012394	0.106586	0.200880	0.750350
**Pyruvic acid**	<0.000001	<0.000001	<0.000001	<0.000001	0.03668	0.14357	<0.000001	0.000004
D-Galactose	>0.999999	0.884575	>0.999999	0.976993	>0.999999	>0.999999	>0.999999	0.990196
**Glucose**	0.000003	0.000078	0.000728	0.012094	0.043091	0.147974	0.391322	0.848327
Glycerol	0.755447	0.792575	0.485028	0.754791	0.622828	0.782485	0.453283	0.873941
Dimethylsulfone	0.008006	0.040129	0.117939	0.388976	0.191493	0.360871	0.071395	0.450682
TG	0.375336	0.558219	0.195411	0.503622	0.040879	0.14357	0.448768	0.873941
Chol	0.407412	0.580412	0.306672	0.595342	0.832481	0.971695	0.559481	0.952374

LDL	0.41845	0.583846	0.57678	0.837059	0.938697	>0.999999	0.502564	0.885331
HDL	0.207444	0.382651	0.41845	0.727325	0.008615	0.106586	0.982785	0.990196
ApoA1	0.563685	0.687289	0.195411	0.503622	0.040879	0.14357	0.914083	0.990196
ApoA2	0.163779	0.335847	0.019767	0.163657	0.675627	0.835237	0.475261	0.885331
ApoB100	0.817504	0.831888	0.644272	0.875507	0.335031	0.488418	0.183978	0.750350
LDL/HDL	0.798845	0.825409	0.260861	0.555661	0.171206	0.339185	0.495603	0.885331
ApoB100/ApoA 1	0.828586	0.836872	0.183573	0.490009	0.040082	0.14357	0.356125	0.834046
ApoB Particles	0.817504	0.831888	0.644272	0.875507	0.335031	0.488418	0.183978	0.750350
VLDL Particles	0.262981	0.428817	0.271363	0.555661	0.027439	0.124708	0.746070	0.990196
IDL Particles	0.500405	0.625113	0.563685	0.834253	0.315946	0.478658	0.812338	0.990196
LDL Particles	0.98466	0.884575	0.758301	0.918516	0.892933	>0.999999	0.156309	0.750350
LDL-1 Particles	0.182269	0.352404	0.847517	0.929166	0.182269	0.352075	0.982785	0.990196
**LDL-2 Particles**	0.000599	0.004771	0.077343	0.307416	0.070931	0.187787	0.268039	0.828731
LDL-3 Particles	0.126084	0.289225	0.904257	0.945309	0.13999	0.292333	0.705270	0.990196
LDL-4 Particles	0.675904	0.762308	0.85159	0.929166	0.309002	0.472793	0.307870	0.831068
LDL-5 Particles	0.223714	0.388172	0.72919	0.918516	0.06558	0.182389	0.214989	0.757462

LDL-6 Particles	0.007513	0.039106	0.847517	0.929166	0.020003	0.106586	0.040076	0.374260
VLDL TG	0.209222	0.382651	0.231218	0.528528	0.020003	0.106586	0.502564	0.885331
IDL TG	0.254776	0.425696	0.315946	0.605482	0.028881	0.127515	0.502564	0.885331
LDL TG	0.070931	0.20869	0.286408	0.575319	0.696818	0.854598	0.156309	0.750350
HDL TG	0.957939	0.884575	0.77682	0.928952	0.832481	0.971695	0.754610	0.990196
VLDL	0.57337	0.692856	0.129755	0.395833	0.035308	0.14357	0.681573	0.990196
IDL	0.647858	0.755872	0.262981	0.555661	0.378001	0.526239	0.845997	0.990196
LDL	0.41845	0.583846	0.57678	0.837059	0.938697	>0.999999	0.502564	0.885331
HDL	0.207444	0.382651	0.41845	0.727325	0.008615	0.106586	0.982785	0.990196
VLDL FC	0.362415	0.544992	0.162279	0.475035	0.018686	0.106586	0.803637	0.990196
IDL FC	0.889124	0.878353	0.180646	0.490009	0.260868	0.438173	0.991786	0.990196
LDL FC	0.145323	0.313227	0.802593	0.929166	0.221821	0.403271	0.329136	0.831068
HDL FC	0.017942	0.075884	0.791415	0.929166	0.001135	0.053504	>0.999999	0.990196
VLDL PL	0.175944	0.35018	0.28866	0.575319	0.016067	0.106586	0.914083	0.990196
IDL PL	0.919607	0.884575	0.165252	0.475035	0.12729	0.283852	0.721554	0.990196
LDL PL	0.700457	0.770731	0.938697	0.971641	0.836313	0.971695	0.249468	0.804138
HDL PL	0.700457	0.770731	0.09541	0.356549	0.010154	0.106586	0.746070	0.990196

HDL ApoA1	0.488209	0.625113	0.209222	0.512698	0.022262	0.111183	>0.999999	0.990196
HDL ApoA2	0.100401	0.261313	0.024444	0.182698	0.904216	>0.999999	0.530646	0.913556
VLDL ApoB	0.262981	0.428817	0.269202	0.555661	0.027078	0.124708	0.746070	0.990196
IDL ApoB	0.491347	0.625113	0.563685	0.834253	0.309015	0.472793	0.812338	0.990196
LDL ApoB	0.973282	0.884575	0.758301	0.918516	0.892933	>0.999999	0.156309	0.750350
VLDL-1 TG	0.385853	0.56152	0.091554	0.35091	0.013571	0.106586	0.287503	0.831068
VLDL-2 TG	0.168281	0.339928	0.452584	0.754791	0.030042	0.128957	0.746070	0.990196
VLDL-3 TG	0.121291	0.289225	0.640685	0.875507	0.071698	0.187787	0.982785	0.990196
VLDL-4 TG	0.059389	0.186925	0.620362	0.874828	0.121291	0.279748	0.329136	0.831068
VLDL-5 TG	0.503443	0.625113	0.904329	0.945309	0.620234	0.782485	0.455718	0.873941
VLDL-1 Chol	>0.999999	0.884575	0.05368	0.286577	0.038928	0.14357	0.345471	0.834046
VLDL-2 Chol	0.911958	0.884575	0.124905	0.388976	0.061413	0.17906	0.704789	0.990196
VLDL-3 Chol	0.407412	0.580412	0.388563	0.699805	0.131021	0.283852	0.922901	0.990196
VLDL-4 Chol	0.534674	0.657843	0.817504	0.929166	0.410133	0.554105	0.423105	0.866223
VLDL-5 Chol	0.711103	0.776135	0.29521	0.580631	0.388482	0.531257	0.754450	0.990196
VLDL-1 FC	0.42966	0.593369	0.074081	0.307416	0.015194	0.106586	0.329136	0.831068
VLDL-2 FC	0.754627	0.792575	0.068605	0.307416	0.014959	0.106586	0.391966	0.848327

VLDL-3 FC	0.647693	0.755872	0.229297	0.528528	0.096382	0.240225	0.650117	0.990196
VLDL-4 FC	0.732783	0.792575	0.233069	0.528528	0.2953	0.472793	0.974194	0.990196
VLDL-5 FC	0.461258	0.612026	0.473075	0.754791	0.934857	>0.999999	0.871388	0.990196
VLDL-1 PL	0.271363	0.432073	0.124843	0.388976	0.014977	0.106586	0.329136	0.831068
VLDL-2 PL	0.131021	0.29554	0.396546	0.705663	0.017694	0.106586	0.746070	0.990196
VLDL-3 PL	0.221849	0.388172	0.441038	0.754791	0.05422	0.167573	0.948389	0.990196
VLDL-4 PL	0.244752	0.419301	0.988485	0.976993	0.168229	0.337617	0.423105	0.866223
VLDL-5 PL	0.973155	0.884575	0.207386	0.512698	0.2527	0.438173	0.837101	0.990196
LDL-1 TG	0.297577	0.468302	0.388572	0.699805	0.851421	0.981866	0.567008	0.954464
LDL-2 TG	0.843728	0.845853	0.485116	0.754791	0.290832	0.472793	0.530646	0.913556
LDL-3 TG	0.754622	0.792575	0.070087	0.307416	0.210913	0.392679	0.034716	0.374260
LDL-4 TG	0.09439	0.250486	0.207445	0.512698	0.503542	0.659427	0.061642	0.411146
LDL-5 TG	0.045502	0.157905	0.668547	0.888377	0.132255	0.283852	0.100633	0.564663
**LDL-6 TG**	0.000181	0.00272	0.35225	0.65818	0.034858	0.14357	0.027516	0.374260
LDL-1 Chol	0.056203	0.181108	0.337445	0.638497	0.256839	0.438173	0.704677	0.990196
**LDL-2 Chol**	0.000306	0.003189	0.049425	0.27852	0.051209	0.161497	0.199058	0.750350
LDL-3 Chol	0.091554	0.247819	0.877749	0.941202	0.146715	0.302292	0.879934	0.990196

LDL-4 Chol	0.958153	0.884575	0.704287	0.918516	0.327832	0.488418	0.374357	0.834046
LDL-5 Chol	0.352299	0.539579	0.455626	0.754791	0.05731	0.17365	0.307870	0.831068
LDL-6 Chol	0.007064	0.039106	0.537925	0.817118	0.016067	0.106586	0.015133	0.327531
LDL-1 FC	0.05483	0.180992	0.761939	0.918516	0.09541	0.240225	>0.999999	0.990196
**LDL-2 FC**	0.00051	0.004598	0.1775	0.490009	0.010002	0.106586	0.249468	0.804138
LDL-3 FC	0.085091	0.235025	0.761948	0.918516	0.059389	0.176489	0.713573	0.990196
LDL-4 FC	>0.999999	0.884575	0.881511	0.941202	0.647698	0.807167	0.323565	0.831068
LDL-5 FC	0.503453	0.625113	0.94252	0.971641	0.286394	0.472793	0.159487	0.750350
LDL-6 FC	0.029998	0.115998	0.372675	0.687747	0.124858	0.283739	0.061642	0.411146
LDL-1 PL	0.214534	0.387133	0.828652	0.929166	0.231218	0.415466	0.973919	0.990196
**LDL-2 PL**	0.000434	0.004191	0.107748	0.383481	0.037549	0.14357	0.374357	0.834046
LDL-3 PL	0.139986	0.310584	0.877749	0.941202	0.150819	0.306658	0.812338	0.990196
LDL-4 PL	0.696817	0.770731	0.847517	0.929166	0.308953	0.472793	0.307870	0.831068
LDL-5 PL	0.269202	0.432073	0.627024	0.875507	0.04543	0.152615	0.231790	0.780358
LDL-6 PL	0.004833	0.029733	0.541174	0.817118	0.0192	0.106586	0.017815	0.334274
LDL-1 ApoB	0.180671	0.352404	0.847517	0.929166	0.180671	0.352075	0.982785	0.990196
**LDL-2 ApoB**	0.000599	0.004771	0.07815	0.307416	0.070931	0.187787	0.268039	0.828731

LDL-3 ApoB	0.122486	0.289225	0.904257	0.945309	0.13999	0.292333	0.705270	0.990196
LDL-4 ApoB	0.675904	0.762308	0.851269	0.929166	0.308952	0.472793	0.302538	0.831068
LDL-5 ApoB	0.223714	0.388172	0.72919	0.918516	0.06558	0.182389	0.214989	0.757462
LDL-6 ApoB	0.007513	0.039106	0.847517	0.929166	0.020003	0.106586	0.040076	0.374260
HDL-1 TG	0.150816	0.314023	0.73998	0.918516	0.383194	0.528705	0.779008	0.990196
HDL-2 TG	0.593401	0.710716	0.467313	0.754791	0.828693	0.971695	0.423105	0.866223
HDL-3 TG	0.252665	0.425696	0.988496	0.976993	0.332553	0.488418	0.746070	0.990196
HDL-4 TG	0.032368	0.121686	0.72919	0.918516	0.116639	0.273094	0.681573	0.990196
HDL-1 Chol	0.03709	0.135669	0.98466	0.976993	0.01077	0.106586	0.681573	0.990196
HDL-2 Chol	0.03943	0.140433	0.71118	0.918516	0.108846	0.258768	0.307870	0.831068
HDL-3 Chol	0.383193	0.56152	0.07172	0.307416	0.441038	0.58251	0.991626	0.990196
HDL-4 Chol	0.027098	0.111135	0.067155	0.307416	0.57678	0.73959	0.153131	0.750350
HDL-1 FC	0.002985	0.020201	0.66161	0.888377	0.000427	0.033006	0.495409	0.885331
HDL-2 FC	0.013761	0.062082	0.236897	0.528528	0.000144	0.022299	>0.999999	0.990196
HDL-3 FC	0.889126	0.878353	0.033172	0.225387	0.047684	0.156778	0.641952	0.990196
HDL-4 FC	0.048874	0.165366	0.005235	0.065205	0.438138	0.58251	0.061642	0.411146
HDL-1 PL	0.028881	0.114964	0.590014	0.839955	0.008486	0.106586	0.914083	0.990196

HDL-2 PL	0.354829	0.539579	0.586606	0.839955	0.260868	0.438173	0.973919	0.990196
HDL-3 PL	0.117808	0.289225	0.028881	0.20558	0.762101	0.921827	0.681573	0.990196
HDL-4 PL	0.003251	0.020951	0.01077	0.107324	0.3574	0.506688	0.100633	0.564663
HDL-1 ApoA1	0.005855	0.034455	0.836477	0.929166	0.004243	0.093665	0.351302	0.834046
HDL-2 ApoA1	0.938697	0.884575	0.099393	0.362373	0.050013	0.16101	0.713573	0.990196
HDL-3 ApoA1	0.124844	0.289225	0.067889	0.307416	0.806301	0.965874	0.713573	0.990196
HDL-4 ApoA1	0.01077	0.052056	0.044985	0.268972	0.344841	0.498021	0.075460	0.457290
HDL-1 ApoA2	0.074868	0.215589	0.449654	0.754791	0.006535	0.106586	0.879934	0.990196
HDL-2 ApoA2	0.627089	0.744476	0.117748	0.388976	0.240768	0.425846	0.626815	0.990196
HDL-3 ApoA2	0.11777	0.289225	0.145311	0.434423	>0.999999	>0.999999	0.905308	0.990196
HDL-4 ApoA2	0.012996	0.060653	0.11548	0.388976	0.372671	0.523535	0.180238	0.750350

Table S2: Results of simple linear regression analysis between participants with overweight BMI (>25 kg/m²) and quantified IVDr parameters.

	R squared	*P* value
Ethanol	0.0007259	0.8527
Trimethylamine-N-oxide	1	
2-Aminobutyric acid	0.007953	0.538
Alanine	0.03185	0.215
Asparagine	0.09254	0.0317
Creatine	0.07695	0.0511
Creatinine	0.0246	0.2767
Glutamic acid	0.06647	0.0707
Glutamine	0.06422	0.0758
Glycine	0.02871	0.2395
Histidine	0.06883	0.0657
Isoleucine	0.003825	0.6696
Leucine	0.008076	0.5348
Lysine	0.0149	0.3984
Methionine	0.02126	0.3123

N,N-Dimethylglycine	0.07675	0.0515
Ornithine	0.1369	0.0082
Phenylalanine	0.01543	0.3901
Proline	0.007971	0.5375
Sarcosine	0.05813	0.0917
Threonine	0.00002088	0.9749
Tyrosine	0.06233	0.0804
Valine	0.005269	0.6164
2-Hydroxybutyric acid	0.001443	0.7934
Acetic acid	0.05847	0.2063
Citric acid	0.04904	0.1222
Formic acid	0.07671	0.0515
Lactic acid	0.02237	0.2998
Succinic acid	0.004826	0.6317
Choline	0.1776	0.0023
2-Oxoglutaric acid	0.0001724	0.9279
3-Hydroxybutyric acid	0.007857	0.5404

Acetoacetic acid	0.00004311	0.9639
Acetone	0.0009377	0.8328
Pyruvic acid	0.1608	0.0047
D-Galactose	1	
Glucose	0.1732	0.0026
Glycerol	0.3255	<0.0001
Dimethylsulfone	0.0438	0.1446
TG	0.01178	0.4531
Chol	0.008578	0.5224
LDL	0.003535	0.6817
HDL	0.04497	0.1393
ApoA1	0.06482	0.0744
ApoA2	0.07829	0.0491
ApoB100	0.009636	0.4977
LDL/HDL	0.01413	0.411
ApoB100/ApoA1	0.03414	0.1989
ApoB Particles	0.00964	0.4976

VLDL Particles	0.02208	0.303
IDL Particles	0.01056	0.4776
LDL Particles	0.004176	0.6557
LDL-1 Particles	0.01051	0.4786
LDL-2 Particles	0.006073	0.5906
LDL-3 Particles	0.002031	0.756
LDL-4 Particles	0.01371	0.4181
LDL-5 Particles	0.0002387	0.9152
LDL-6 Particles	0.0001177	0.9404
VLDL TG	0.007378	0.5531
IDL TG	0.006965	0.5645
LDL TG	0.002449	0.7329
HDL TG	0.000611	0.8647
VLDL	0.03455	0.1962
IDL	0.02609	0.2625
LDL	0.003535	0.6817
HDL	0.04497	0.1393

VLDL FC	0.02891	0.2378
IDL FC	0.02931	0.2345
LDL FC	0.001319	0.8023
HDL FC	0.0191	0.3385
VLDL PL	0.01232	0.4429
IDL PL	0.01344	0.4227
LDL PL	0.0009651	0.8304
HDL PL	0.05688	0.0953
HDL ApoA1	0.06358	0.0773
HDL ApoA2	0.08	0.0466
VLDL ApoB	0.02207	0.3032
IDL ApoB	0.01064	0.476
LDL ApoB	0.004178	0.6556
VLDL-1 TG	0.007358	0.5536
VLDL-2 TG	0.002085	0.7528
VLDL-3 TG	0.005992	0.5931
VLDL-4 TG	0.003148	0.6987

VLDL-5 TG	0.00008209	0.9502
VLDL-1 Chol	0.0261	0.2624
VLDL-2 Chol	0.03134	0.2187
VLDL-3 Chol	0.0288	0.2387
VLDL-4 Chol	0.02565	0.2665
VLDL-5 Chol	0.03786	0.1757
VLDL-1 FC	0.01011	0.4872
VLDL-2 FC	0.04278	0.1495
VLDL-3 FC	0.02788	0.2465
VLDL-4 FC	0.04687	0.131
VLDL-5 FC	0.04496	0.1393
VLDL-1 PL	0.006068	0.5908
VLDL-2 PL	0.004634	0.6386
VLDL-3 PL	0.009385	0.5033
VLDL-4 PL	0.0128	0.4341
VLDL-5 PL	0.02055	0.3206
LDL-1 TG	0.008541	0.5233

LDL-2 TG	0.0003857	0.8923
LDL-3 TG	0.0002156	0.9194
LDL-4 TG	0.01462	0.4029
LDL-5 TG	0.00459	0.6402
LDL-6 TG	0.0007142	0.8538
LDL-1 Chol	0.02627	0.2607
LDL-2 Chol	0.006353	0.5822
LDL-3 Chol	0.006062	0.591
LDL-4 Chol	0.01649	0.3742
LDL-5 Chol	0.0009225	0.8342
LDL-6 Chol	0.001854	0.7666
LDL-1 FC	0.001265	0.8063
LDL-2 FC	0.001889	0.7644
LDL-3 FC	0.0004074	0.8893
LDL-4 FC	0.004454	0.6452
LDL-5 FC	0.0002951	0.9058
LDL-6 FC	0.02671	0.2568

LDL-1 PL	0.01359	0.4201
LDL-2 PL	0.003256	0.6939
LDL-3 PL	0.002539	0.7282
LDL-4 PL	0.01294	0.4315
LDL-5 PL	0.0001075	0.943
LDL-6 PL	0.00489	0.6294
LDL-1 ApoB	0.01052	0.4784
LDL-2 ApoB	0.006078	0.5905
LDL-3 ApoB	0.002041	0.7554
LDL-4 ApoB	0.01371	0.418
LDL-5 ApoB	0.0002393	0.9151
LDL-6 ApoB	0.000117	0.9406
HDL-1 TG	0.0007701	0.8483
HDL-2 TG	0.00000923	0.9833
HDL-3 TG	0.0004856	0.8793
HDL-4 TG	0.002196	0.7466
HDL-1 Chol	0.01443	0.406

HDL-2 Chol	0.02102	0.3152
HDL-3 Chol	0.08203	0.0438
HDL-4 Chol	0.02071	0.3187
HDL-1 FC	0.007878	0.5399
HDL-2 FC	0.05294	0.108
HDL-3 FC	0.08496	0.04
HDL-4 FC	0.02478	0.275
HDL-1 PL	0.01497	0.3973
HDL-2 PL	0.0376	0.1772
HDL-3 PL	0.1147	0.0161
HDL-4 PL	0.04618	0.134
HDL-1 ApoA1	0.006009	0.5926
HDL-2 ApoA1	0.0452	0.1383
HDL-3 ApoA1	0.08876	0.0356
HDL-4 ApoA1	0.03644	0.1842
HDL-1 ApoA2	0.006419	0.5802
HDL-2 ApoA2	0.03796	0.1752

HDL-3 ApoA2	0.04509	0.1387
HDL-4 ApoA2	0.01357	0.4204
